# Impact of Taste on Food Choices in Adolescence—Systematic Review and Meta-Analysis

**DOI:** 10.3390/nu12071985

**Published:** 2020-07-03

**Authors:** Areej O. Bawajeeh, Salwa A. Albar, Huifeng Zhang, Michael A. Zulyniak, Charlotte E. L. Evans, Janet E. Cade

**Affiliations:** 1 Nutritional Epidemiology Group, School of Food Science and Nutrition, University of Leeds, Leeds LS2 9JT, UK; fshz@leeds.ac.uk (H.Z.); M.A.Zulyniak@leeds.ac.uk (M.A.Z.); C.E.L.Evans@leeds.ac.uk (C.E.L.E.); J.E.Cade@leeds.ac.uk (J.E.C.); 2 School of Food Science and Nutrition, King Abdulaziz University, PO Box 42807,21551, Jeddah 21589, Saudi Arabia; sabar.c@sfda.gov.sa; 3 Saudi Food and Drug Authority, Northern Ring Branch Road, Dist. Unit Number: 1 4904 Dist., Riyadh 6336, Saudi Arabia

**Keywords:** taste, sweet, sour, bitter, umami, genetics, genotype, phenotype, adolescents, food choices, food intakes

## Abstract

Studies of adults report that perceived taste affects food choices and intake, which in turn may have an impact on health. However, corresponding evidence on adolescents is limited. Our aim was to summarize current evidence of the impact of taste perception on food choice preferences or dietary intakes among adolescents (mean age 10–19.9 years). Systematic searches identified 13 papers, 12 cross-sectional and one cohort study published between 1 January 2000 to 20 February 2020 assessing the impact of taste (using phenotypic and/or genotypic markers) on food choices in adolescents without any disease conditions. Qualitative assessment in the current review indicated that individuals sensitive to bitter tastes often have a lower preference of bitter-tasting food and higher preference for sweet-tasting food. A meta-analysis of three studies on bitter-taste sensitivity revealed no difference in preference for bitter-tasting vegetables between bitter tasters and non-tasters (standardized mean difference (SMD) = 0.04; 95% CI: −0.18, 0.26; *p* = 0.72). Overall, a limited number of studies were available for review. As a result, we report no clear relationship between taste perception and food choices or intake in adolescents. More studies are needed to evaluate the link between adolescents’ taste perceptions and dietary intake.

## 1. Introduction

Taste perceptions differ between individuals due to genetics, culture, ethnicity, personal, and environmental factors. The extent to which adults perceive taste has been well defined as a determinant of dietary intake [1] and food choices [1,2,3,4].

Studies have reported differences in individual intensity perception and preferences for all tastes [2], bitter [5], sour [6], sweet [7], salt [8], and umami [9]. Genotype and phenotype methods for taste assessment have been used to identify an individual’s taste characteristics [10]. The human taste phenotype is based on reactions of chemical substances in food with taste receptors located on the tongue [1] encoded by different genes [1,11,12]. Once those chemical stimuli are mixed with the saliva and digestive enzymes, the taste is detected [13]. Based on that, individuals are classified as tasters (those who can perceive/detect taste at low concentrations) or non-tasters (barely perceive/detect taste or not at all) [14]. Salty and sour tastes are delivered through ion channels, and specific genetic variants within taste receptor genes can also be used to stratify individuals as tasters or non-tasters [15]. G-coupled protein receptors T1R2, T1R3, and T1R38 encoded by *TAS1R2*/*TAS1R3* and *TAS1R1*/*TAS1R3*, are involved in perceiving sweet and umami tastes [12,16]. *TAS2R38* is the commonly studied gene responsible for perceiving bitter taste, and different single-nucleotide polymorphisms (SNPs) within this receptor are responsible for different bitter perceptions [12].

The taste of food was reported as an important factor in food choice in adolescence, which is a critical phase of human development [17], transitioning from childhood to adulthood [18]. Thus, healthy eating and good nutrition are required during this age to meet growth needs [19]. However, adolescent eating habit is often characterized by high-calorie-dense foods primarily sourced from fats and sugars rather than fruit and vegetables [20,21,22,23].

It has been reported that younger-aged individuals have a higher preference for high concentrations of sugar and are also more sensitive to the taste of bitter compared to adults [12,24]. This may suggest an association between taste sensitivity and taste preference, where individuals with high bitter-taste sensitivity may reflect a low preference for these foods [25]. In a couple of studies comparing taste preference and dietary consumption between adults and younger individuals, adult bitter tasters reported a higher preference and consumption for bitter-tasting vegetables compared to younger individuals who were bitter tasters [12]. In contrast, when sweet taste was investigated, the opposite was noted, where younger-age participants showed higher preference for sweet-tasting food compared to adults [24]. These differences may be explained by adults’ cognitive attitude and awareness of health-benefits of bitter-tasting foods [1], differences in hedonistic reward, and self-control or due to reduction in sweet taste perception with age. 

Links between taste preferences and food intake may be associated with future health [1,3]. For example, an adult study identified a possible increased risk of colon cancer in bitter-taster men associated with low vegetable consumption [26]. Concerning adolescents, dietary behaviour of high sugar and low vegetable consumption may be a leading cause of adolescent obesity [23], which raises a concern with a projection of 2.7 billion overweight and 1 billion obese adults by 2025 [27]. Thus, because adolescence is a critical phase of development, transitioning between childhood and adulthood [18], the purpose of this systematic review is to summarize the evidence linking taste perception (genotype and phenotype), to food choice among adolescents.

## 2. Materials and Methods 

### 2.1. Search Strategy

A protocol was designed and agreed on by all authors, the review protocol was published in PROSPERO with the registration number: CRD42019134088 [28]. 

The following databases were searched: Ovid MEDLINE In-Process, Embase, Web of Science, CINAHL, PsycINFO, and CAB Abstracts. Searching in MEDLINE, Embase, and PsycINFO included combinations of the research question concepts’ terms, phrases, and medical subject headings (MeSH) as follows: (“tast*” or “sweet*” or “sour*” or “salt* ”or “bitter*” or “fat*” or “savo?r*” or “cream* ”or “PROP” or “PTC” or “pungent*” or “astringent*” or “tast* adj3 fat” or “Taste/ or Taste Threshold/ or Taste Perception/” or “tast* adj3 cream*”) AND (“adolescent*” or “child*” or “young adult*” or “youth*” or “secondary school*” or “high school*” or “Adolescent/”) AND (“gene*” or “genetic*” or “phenotype*” or “ genotype*” or “Genes/” or “Genetics/”) AND (“food preferenc*” or “ food lik*” or “food choic*” or “food intak*” or “FFQ” or “24-hour recall” or “Food Preferences/ ” or “appetite*”). These keywords and phrases were adapted to be used with other databases when medical subject headings were not available such as in Web of Science.

### 2.2. Inclusion and Exclusion Criteria

The searches were applied to the period from 1 January 2000 to 1 January 2019, this period is appropriate due to the lack of publication prior to it. All primary-type studies of human subjects published in English were considered. The search was re-run from 1 January 2019 to 20 February 2020 for potential new studies. Studies were included if they were in adolescents without a history of health-related issue or diseases and aged 13–18 years with a population mean age between 10–19 years. This mean age is based on the WHO definition of adolescents [29], we did not include younger ages of 10–12 years since these studies would often have a mean age below the WHO definition. To be eligible for inclusion, studies needed to include taste assessment for either genotype or phenotype as well as outcomes relating to food choices and intake measurements such as a 24-h diet recall, food frequency questionnaire (FFQ), or a food preference questionnaire. To be eligible for a quantitative study, studies with more than two results on taste perception and food preference/intake for the same taste and taste test used (phenotype or genotype) were included in a meta-analysis.

### 2.3. Study Selection

Titles and abstracts were independently screened in duplicate by four members of the study team (A.B., S.A., M.A.Z., and J.C.). Any disagreements between screeners were evaluated and decided by the fifth member (C.E.). Full-text articles were independently screened in duplicate by four members of the study team. Any disagreements were resolved by discussion.

### 2.4. Data Extraction

Data were extracted independently in duplicate. All data were extracted into Microsoft Excel. Any disagreements were resolved by discussion. Extracted data for the narrative synthesis included demographic information, study design, anthropometric data, methods of testing and measuring taste perception, as well as food intake and studies’ results. Only studies on taste perception and preference of bitter-tasting vegetables were able to be meta-analysed, so the effect sizes (means and measures of variance) from these studies were extracted. This was due to the very limited number of studies on other tastes, and it was not possible to include these in a meta-analysis. Bitter-tasting vegetables were classified according to definitions in other studies [30,31,32,33].

### 2.5. Quality Assessment of Studies

Quality assessment of included studies was carried out in duplicate, independently using the Newcastle–Ottawa Scale for observational studies [34,35]. The scale utilizes a “star system” of points relating to selection of study groups, comparability of groups, and ascertainment of exposure and outcome with a total maximum of 10 points. A study with ≥5 points was considered a high-quality paper [36].

### 2.6. Statistical Analysis

One study reported standard error of the mean (SEM), this was used to calculate the standard deviation (SD). Since two of the studies reported separate results for multiple types of bitter vegetables for tasters and non-tasters, we pooled each study’s results into one combined bitter vegetable grouping for both taster groups using Stata software [37] to be used in the meta-analysis.

Meta-analysis was carried out using RevMan version 5.3 [38]. Due to anticipated heterogeneity between measures of taste preference and taste phenotype between studies and study populations, a random-effects model was used to evaluate mean effect size. Standardized mean difference (SMD) was calculated by dividing the mean difference in each study by its standard deviation [39]. The SMD was used as preference scales were not directly comparable to estimate differences in bitter-taste vegetable preference between bitter tasters and non-tasters.

## 3. Results

### 3.1. Systematic Search 

**Figure 1:**
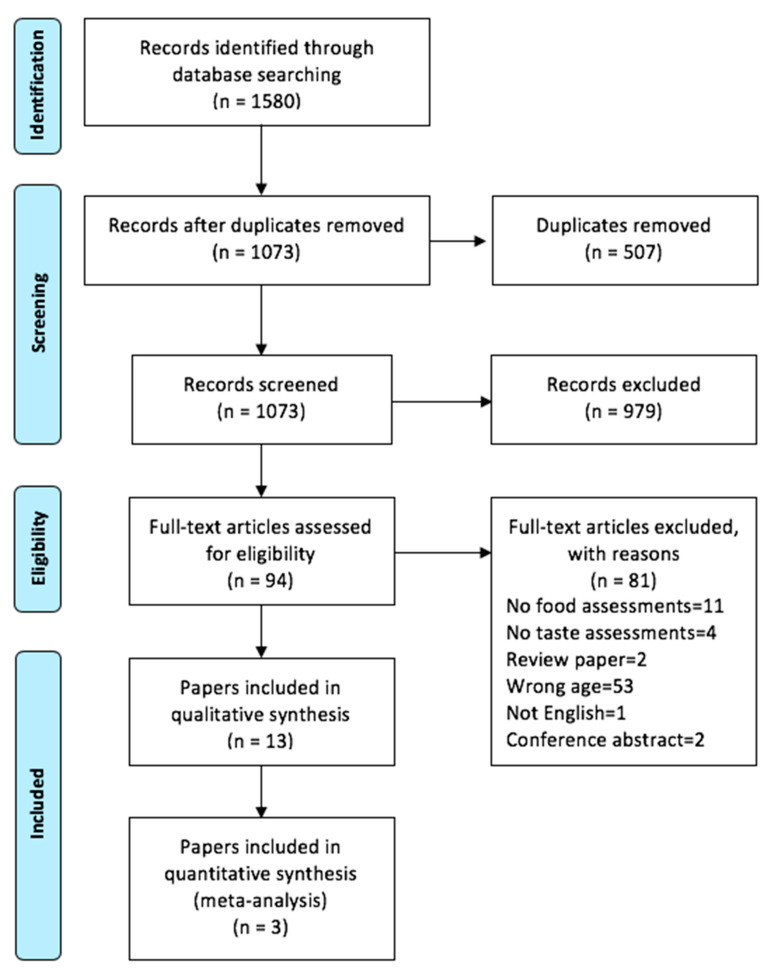
Flow diagram indicating number of studies.

Our search identified a total of 1580 potential articles, including 507 duplicates. The remaining 1073 references went through titles and abstracts screening, and of these, 94 potential articles met our criteria for full-text screening. At this stage, 81 studies were removed, which resulted in a final number of 10 studies (9 cross-sectional and 1 follow-up study) published in 13 papers. Those 13 papers were included in the qualitative synthesis, of which 3 were included in the quantitative meta-analysis since the measures and outcomes were consistent. The re-running of the searches retrieved no additional relevant papers. The PRISMA flow diagram, with reasons for exclusion, is shown in Figure 1, the majority were excluded because they could not be considered adolescent-based studies. The quality of the included papers ranged from 3 to 7 (Table 1) with an average of 5.7 points. Two papers had low quality while 11 showed high quality based on the ≥5 points categorization.

### 3.2. Study Characteristics 

Table 2 provides a summary of the descriptive characteristics of the included studies. The total number of participants was 2229 (females = 1281, males = 933, and not reported = 15), of which 1481 participants had completed taste test measurements (genotype and/or phenotype) and food preference or food intake evaluations. Participants were from different geographic regions, which were the Philippines, India, Japan, Ireland, and USA.

Taste perception was assessed in all 13 papers: 5 papers conducted taste phenotype measures [40,41,42,47,52], 3 papers conducted taste genotype measures [45,50,51], and 5 papers measures both phenotype and genotype [43,44,46,48,49].

Bitter was the most studied taste in 9 papers. 6 papers used 6-n-propylthiouracil (PROP) [40,41,43,44,48,49] and 1 used phenylthiocarbamide (PTC) [52] to test bitter taste, while 6 papers genotyped the following single-nucleotide polymorphisms (SNPs): rs713598, rs1726866, and rs10246939 in the gene *TAS2R38* [43,44,45,46,48,49].

Five papers studied the sweet-taste phenotype: 4 used sucrose [42,43,44,46], one used fructose solution and blueberry fruit [47], and 2 papers explored genotype for sweet-taste relating to genes *TAS1R2* (rs9701796; rs35874116) [51]; *TAS1R3* (rs35744813); and *GNAT3* (rs7792845) [46]. The fat-taste gene, *CD36*, (rs1761667) was studied in one paper [50], while no studies reported on umami and sour tastes as seen in Table S1 in the Supplementary Materials. Table S2 in the Supplementary Materials illustrates genes and SNPs associated with each taste included in the current review.

Food preference and food intake were assessed in a variety of ways across studies, including food preference and behaviour questionnaires, food record, 24-h dietary recall, and FFQ. The variation of food included (Table S3 in the Supplementary Materials) limited the number of meta-analyses that could be undertaken.

### 3.3. Qualitative Summary of Findings

#### 3.3.1. Bitter 

Generally, the proportion of bitter tasters was higher than that of non-tasters within the included cohorts. In Filipinos adolescents, 93% were tasters and 7% non-tasters [40]; and in Indian adolescents, 80% were classified as tasters and 20% non-tasters [52]. Around two-thirds of adolescents from South-eastern USA were tasters (68%) against 32% non-tasters [41]. White Caucasian and Irish groups were classified as 75% tasters and 25% non-tasters [43,49].

Perceived bitterness was negatively correlated with preference for bitter-tasting food such as dark chocolate (*r* = −0.155, *p* = 0.035) and chili peppers (*r* = −0.144, *p* = 0.046) [41]. A similar association was reported with bitter-tasting vegetables where bitter-sensitive individuals (tasters) reported lower preference for cruciferous vegetables such as cabbage [52] and broccoli [43] than individuals who were non-tasters. Similarly, the less sensitive AVI homozygous haplotypes carriers (non-tasters) and PROP non-tasters, had an increased liking for brussels sprouts and cauliflower [43]. 

Studies also reported that perceived bitterness was associated with other tastes. Joseph et al., 2016, found more added sugar in the diet of individuals carrying the bitter-sensitive genotype in *TAS2R38*, as well as an increased preference for sweet-tasting food [46]. Likewise, PTC tasters reported a higher preference for sweet-tasting food [52]. Bitter tasters were also observed to have higher preference for salty and sour condiments and high-protein foods known to have an umami taste [40]. Table S4 in the Supplementary Materials illustrates the liked and disliked food based on bitter-taste sensitivity as reported in the included studies. 

As for nutrient intakes, a higher mean intake of energy was found in PROP medium tasters (1952 ± 666 kcal) and supertasters (1851 ± 656 kcal) compared to non-tasters (1620 ± 364 kcal), (*p* < 0.05) in a study of 120 Filipino adolescents [40]. However, Inoue et.al, 2013, found the opposite in a smaller study (*n* = 47) of older Japanese college students, reporting significantly higher intakes of energy in AI/AI haplotype carriers (non-tasters) comparing to PV/PV and PV/AI haplotype carriers (tasters) (AI/AI carriers = 1742 ± 216 kcal; PV/PV and PV/AI = 1512 ± 259 kcal, *p* = 0.02). The same pattern was noted with carbohydrate intakes (AI/AI carriers = 254.7 ± 34.4 kcal; PV/PV and PV/AI = 217.3 ± 37.4 kcal, *p* = 0.01) [45].

#### 3.3.2. Sweet

Concerning sweet taste, participants with a high sweet threshold were found to prefer food items with higher sugar content. In one study, researchers examined sweet-taste preference using blueberries at different harvest times, which has an impact on the sugar content of fruits. This influenced participant liking and preference where they preferred the sweetest berries [47]. Similarly, participants in another study were asked to taste a flavoured beverage (orange Kool-Aid^®^ drink) with four different sugar concentrations where participants with a high sweet threshold reported a higher preference for the drink with the highest sugar concentration compared to other concentrations [42].

Regarding sweet-taste genotype, obese individuals with an allelic variant in the SNP rs9701796 in the sweet-related gene, *TAS1R2*, reported a higher intake of sweet chocolate powder [51]. In contrast, in another sweet-related gene, *TAS1R3*, and *GNAT3* genes were not associated with sucrose taste threshold or intake of sugar [46].

#### 3.3.3. Fatty 

Although fat is not traditionally recognized as a primary taste [53], there was one cross-sectional study exploring fat-related genes included in this review, the researchers studied the effect of the genetic variation in the *CD36* gene on food intake in both obese and normal-weight adolescents. Statistical differences in dietary intakes were noted in the obese participants but not for normal-weight participants. Obese participants with an allelic variant in rs1761667 of the *CD36* gene had a significantly lower intake of fatty food (266.0 g/d) compared to those with homozygous alleles (343.2 g/d) (*p* < 0.01), which also translated to a lower intake of total fat (49.2 versus 62.4 g/d; *p* = 0.01). More specifically, the total intake of monounsaturated and polyunsaturated fatty acids was significantly less (*p* = 0.01) but not that of saturated fatty acids. Additionally, genetic variation in the *CD36* gene was found to impact sugar intake, where obese participants with the allelic variant also had lower intake of sugar compared to the homozygous group (*p* = 0.01) [50].

#### 3.3.4. Other Tastes 

There were no papers on the perception of salty, sour, and umami tastes and food choices and intakes in adolescents.

### 3.4. Meta-Analysis 

Three studies identifying the bitter-taste phenotype in relation to preference for bitter-taste vegetables were included in the meta-analysis, one of the studies reported females and males separately, providing four effect sizes. Bitter-tasting vegetables included broccoli, cauliflower, sprouts, cabbage, and bitter gourd. The use of different food preference scales (i.e., five-points [41,43] and nine-points [40]) required the use of the standardized mean difference (SMD).

**Figure 2:**
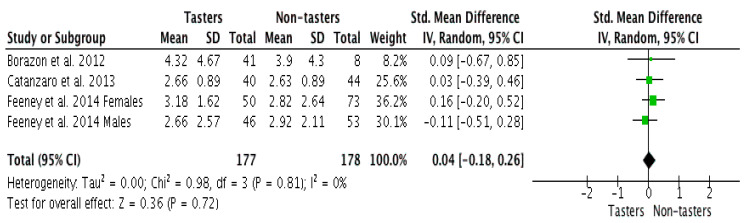
Pooled estimate of bitter-taste vegetable preference between tasters and non-tasters.

The meta-analysis (Figure 2) shows no clear difference in adolescents’ preference for bitter-tasting vegetables between bitter tasters and non-tasters (SMD = 0.04; 95% CI: −0.18, 0.26; *p* = 0.72). A low level of heterogeneity was observed in our analysis denoted by I-squared (0%) and chi-squared (*p* = 0.98). With only four effect sizes, we also ran a fixed-effects model, also with low I-squared (0%), and reporting the same effect size.

## 4. Discussion

This is the first systematic review and meta-analysis in adolescents to investigate the impact of taste perception on food choices. A number of studies on adolescents dietary behaviour observed a calorie-dense diet full of sweet-source food [54,55,56] and low in vegetables [54,57]. The taste of food was reported as an important factor in adolescents’ food choices [20,58,59,60]. Previous reviews in adults have reported potential effects of taste perceptions (genotype and phenotype) on food choices [1,3]. However, given the fact that taste perceptions change with age [2], this suggests that evidence obtained from adult studies linking perceived taste, food choices, and intakes, may not directly translate to younger populations. Thus, taste may have an impact on adolescent eating and food choices, however, the evidence base is limited and more studies to understand adolescences’ taste perceptions and dietary pattern are needed in order to overcome any prediction of increased health risk in adulthood.

Bitter taste was the most studied for its impact on food preference and intake [40,41,43,44,45,46,48,49,52]; followed by sweet taste [42,43,44,46,47,51]. Only one study reported on fat taste [50], while no studies in adolescents reported on umami and sour tastes. Taste testing approaches varied across studies in terms of components used: with PROP [40,41,43,44,48,49] and PTC [52] for bitter taste; and sucrose [42,43,44,46], fructose solutions, and real food using blueberries for sweet taste [47]. Furthermore, taste phenotyping methods and assessment for dietary preference and intake also differed between studies as did the food examined (Table S3). Food studied was either based on food being commonly consumed for the population studied [40,52], or as reported by participants through a 24-h diet recall [48,51]. Furthermore, some foods were studied because they are often avoided for their bitterness such as cruciferous vegetables (e.g., broccoli, cauliflower, and cabbage) [1,61,62]. While researchers have focused on studying single food items in relation to taste, questions may arise regarding composite food and complex dishes, which involve multiple combined tastes [63]. There is a need for understanding taste profiles based on dietary intakes at national and global levels to support our interpretation of relationships between food choices and health outcomes [64].

Humans’ PROP/PTC bitter sensitivity has been widely studied, and the sensitivity to these thiourea compounds’ bitterness may be reflected in dietary behaviour [4,31,32,65]. It has been observed that increased sensitivity could result in dietary behaviour that is low in vegetables [65], especially, bitter-tasting vegetables such as cruciferous vegetables known for their content of health-related bioactive compounds [53,56]. However, findings are inconsistent [31,66]. In adults, an inverse relationship was reported between bitter sensitivity and preference of bitter-tasting food such as coffee, dark chocolate, green tea, and brassica vegetables [1,4]. Likewise, as found in our qualitative assessment, perceived bitterness among PROP and PTC adolescent tasters were reported to be negatively, albeit weakly associated with the preference of bitter-tasting foods [41,52], while individuals who were less sensitive to PROP and carrying AVI/AVI haplotypes known as non-tasters, reported higher preference for bitter-tasting vegetable [43]. However, our meta-analysis with only three studies did not show any significant association between bitter-tasting phenotype and bitter vegetable preferences, which may be due to the limited number of studies and sample sizes available for inclusion. 

Even though *TAS1R3* and *GNAT3* were not related to sweet perception [46] in this review, a third sweet-related gene, *TAS1R2*, has been observed to be linked with sweet taste threshold and consumption of sweet food in individuals with obesity compared to individuals with normal weight. This difference in sweet detection and consumption is thought to be related to the leptin level, which increases the threshold to sweet taste in individuals with obesity [51]. Different results in sweet perception in relation to sweet-related genes may depend on the different genes studied, which would support the need for more studies in this area.

Concerning the concordance between phenotype and genotype classifications, this was only mentioned in two studies [43,46]. Regarding bitter-taste, individuals who were classified as less sensitive based on both phenotype and genotype (R^2^ = 0.17, *p* = 0.035) had a higher preference for bitter-tasting vegetables than those who were more sensitive [43]. This phenotype–genotype relationship has also been shown in studies with adults [12,67]. On the other hand, sucrose thresholds were reported to be linked with two SNPs, rs1726866 and rs10246939, in the bitter-related gene, *TAS2R38*, (*p* = 0.01; *p* = 0.05) rather than in the sweet-related gene *TAS1R3* (*p* = 0.36) confirmed by observing more added sugar in the diets of adolescents with high bitter sensitivity [46]. Thus, phenotype–genotype relationship in terms of sweet taste may not be consistent. This is probably emphasizing the difficulty of separating tastes. For instance, sweet has been described to have a “masking effect” on bitter perception [61] and to suppress its perception [63]. Another point is the examined gene/SNPs as different results were reported with *TAS1R3* and *TAS1R2* [46,51].

Perception of one taste appears to be related to other tastes. Bitter and sweet tastes were found to be interrelated [1,61] this was noted as adolescents sensitive to the bitter taste reported lower preference for bitter-tasting vegetables but had a higher preference for sweet-tasting food [46]. This was found in children but not in adults [68], which may in part be explained by the impact of age and cognitive behaviours in adults’ taste perception and food intake [1,68]. In one study in this review, bitter tasters reported their preference for sour, salty, and umami tastes [40]. This is probably due to a taste–taste interaction and the effect of enhancing/suppressing of taste receptors when compounds in foods interact [61,63], where salt and sour were found to have a suppressive effect on perceiving bitterness [63]. However, more research is needed to understand these interactions of tastes in adolescents affecting their dietary behaviour and eating pattern rather than just measuring their taste perception and food preference.

The association between taste and nutrient intakes was inconsistent. In one study, PROP tasters were reported to consume more energy-dense food and have higher daily intake of total energy and carbohydrate [40]. While another study reported the opposite, where the bitter-related genotype AI homozygous (non-taster) individuals had a higher intake of total energy and carbohydrate than other taster groups [45]. This variation may be related to the different ethnic groups in the two studies where variations in factors such as genetic predispositions, environment, and culture in relation to food exposure and beliefs may all influence intake [57,69,70]. Additionally, results may be inconsistent due to the different approaches to testing taste with phenotype versus genotype, or participants’ age [68], where participants recruited by Inoue et al. (2013) were older by an average of 4–5 years. 

The present review study has strengths and limitations. This review is the first to study the associations between taste and food choices in adolescents. The protocol was published in PROSPERO [28]. Additionally, we included both phenotype and genotype measurements for all taste qualities. The search strategy focused on searching for adolescents 13–18 years of age, so we may have missed studies that focused on 10–12 year olds, who are also defined as adolescents by the WHO. However, we did not include this age group as the mean age would likely be below our inclusion criteria. Methods for measuring exposures and outcomes as well as the food items studied varied between the studies. As a result, this limited the studies suitable for meta-analysis. Moreover, types of studies were limited to cross-sectional studies and one follow-up study, and the sample size in some of the included studies was small, which could influence the validity of results.

## 5. Conclusions

Differences in phenotype or genotype may affect taste perceptions and influence food intake preferences in adolescents. Our qualitative assessment of previous studies indicated that bitter-sensitive individuals may have a lower preference for bitter-tasting food and higher preference for sweet-tasting food, though findings were inconsistent. Meta-analysis showed no association between bitter-taste phenotype and preference of bitter-tasting vegetables. However, this lack of association may be due to the limited number of studies included, rather than demonstrating a true lack of association. Thus, more studies are needed to understand (i) how taste perceptions and dietary habits develop in adolescence, and (ii) how strongly these habits predict health and disease risk in adulthood. More evidence will help in understanding the strength of the relationship between taste perception and food choices. Understanding how tastes affect adolescents’ food choices can help the food industry and care providers to offer healthier food options.

## Figures and Tables

**Table 1:** Quality assessment of included studies.

Criteria/Studies	[40]	[41]	[42]	[43]	[44]	[45]	[46]	[47]	[48]	[49]	[50]	[51]	[52]
Study Design	Cross-Sectional	Cross-Sectional	Cross-Sectional	Cross-Sectional	Cross-Sectional	Cross-Sectional	Cross-Sectional	Cross-Sectional	Cross-Sectional	Follow-Up	Cross-Sectional	Cross-Sectional	Cross-Sectional
**Representativeness^1^**	1	0	1	1	1	0	1	1	1	1	1	1	0
**Sample Size ^2^**	1	0	1	1	1	0	1	0	1	0	0	1	1
**Non-Respondents ^3^**	0	1	1	1	1	0	0	0	1	1	0	1	0
**Exposure ^4^**	1	1	0	1	1	1	1	0	1	1	1	1	1
**Comparability ^5^**	1	0	1	1	1	1	1	1	1	1	1	1	1
**Outcome ^6^**	1	1	1	1	1	2	2	0	1	2	1	1	1
**Statistical Test ^7^**	0	1	1	1	1	1	1	1	1	1	1	1	1
**Total Scores**	5	4	6	7	7	5	7	3	7	7	5	7	5

**^1^** Whether the samples were representative and whether they were chosen randomly or not. **^2^** Whether the sampling was justified and satisfactory. **^3^** Whether the non-respondents characteristics and response rate were mentioned and whether the response rate was satisfactory or not. **^4^** Whether the exposure tool was valid or not. **^5^** Whether confounding factors were controlled. **^6^** Method of assessing the outcome. **^7^** Whether the statistical test used was clearly described and appropriate.

**Table 2:** Characteristics of the studies using phenotype and genotypes taste tests (separately and in combination) included in the current systematic review/meta-analysis.

Study	Study Design/Year	Location/Ethnicity	Population characteristics		Study Measurements			Study Outcomes
			Sample	Age (Years)	Taste Studied	Taste Test	Dietary Assessments	
**Phenotype Taste Test**								
* [40]	Cross-sectional 2012	Philippine/Filipino	120 (60 F, 60 M)	13–17 (Mean = 15)	Bitter	3-PROP/3 NaCl	3-day food record and Food preferences	Significant high preference in supertasters for the condiments ** (*p* < 0.05) Positive correlation between PROP tasters and bacon, fried chicken, dried herring, mussels, boiled pork, shrimps, and rice Tasters had higher energy intake than non-tasters
* [41]	Cross-sectional 2013	South-eastern USA/Ethnicity NR	139 (76 F, 48 M) (15 NR)	18–37 (Mean = 19.7)	Bitter	3-PROP/3 NaCl	Food preference questionnaire	Negative correlations between PROP tasters and dark chocolate, *r* = −0.155 (*p* = 0.035) and chili peppers, *r* = −0.144 (*p* =.046), but not bitter vegetables *r* = 0.062 (*p* = 0.235)
[52]	Cross-sectional 2014	India/Indian	210 F	11–18	Bitter	14 PTC solutions	Unstructured questionnaire for last 24 h	Negative correlations between PTC threshold and preference of bitter-tasting foods (*r* = −0.13, *p* = 0.05; raw cabbage *r* = −0.15, *p* = 0.03) Significant positive correlation of PTC TSN with sweet-tasting food (*r* = 0.13, *p* = 0.05)
[42]	Cross-sectional 2009	USA/2 Alaskan Native, 4 American Indian, 14 Asian/Pacific Islander, 31 Black, Non-Hispanic, 11 Hispanic, 73 White, Non-Hispanic, 8 others	143 (65 F, 78 M)	11–15 (Mean = 13.5)	Sweet	6-sucrose solutions 4 orange Kool-Aid^®^ in different concentrations	Dutch Eating Behaviours Questionnaire	No impact found in eating behaviour based on the hedonic of sucrose Individuals with high sugar preference ranked Kool-Aid^®^ with the most sugar concentration (30% sucrose) as best, while individuals with low sugar preference ranked the same concentration of Kool-Aid^®^ as the worst
[47]	Cross-sectional 2017	USA/diverse ethnicity	49 (28 F, 21 M)	6–16 (Mean = 11.9)	Sweet	3 different harvest blueberries 5-fructose solutions	Automated Self-administered 24-h recall system	Significant preference for the sweetest-tasting blueberry (Keecrisp) during the 1st harvest. Preference changed to the other blueberry types (Arcadia and Kestrel) as being sweeter than Keecrisp for the 2nd harvest
**Genotype taste test**								
[45]	Cross-sectional 2013	Japan/Japanese	87 F	18–22	Bitter	*TAS2R38* (rs713598 and rs10246939)	3-day food recording	Higher intake of energy (*p* = 0.02) and carbohydrate (*p* = 0.01) in AI/AI carriers comparing to PV/PV and PV/AI carriers. Vegetable and dairy product intake did not differ among the three groups
[50]	Cross-sectional 2017	Brazil/Brazilian	580	7–18 (Mean = 12.2) obese (Mean = 10.4) normal weight	Fat	*CD36* (rs1761667)	2 24-h food recalls	Significant decreased intake of total fat (*p* = 0.01), polyunsaturated and monounsaturated fatty acids, total sugars (*p* = 0.01), fatty foods (*p* < 0.001), and vegetable oils (*p* = 0.02) in obese subjects carrying A allele of rs1761667 in *CD36* gene
[51]	Cross-sectional 2018	Brazil/Brazilian	648 (303 F, 345 M)	7–18 (Mean = 12.2) obese and (Mean = 10.4) normal weight	Sweet	*TAS1R2* (rs9701796 and rs35874116)	2 24-h food recalls	Significant high intake of the sweet chocolate powder in obese subjects with different allele carriers *p* = 0.04 Significant high intake of MUFA (g and %) *p* = 0.04 in obese subjects carrying serine allele in rs9701796 in *TAS1R2* gene Significant low intake of dietary fibre *p* = 0.002 in obese subjects carrying valine allele in rs35874116 in *TAS1R2* gene
**Phenotypes and genotypes taste tests**								
* [43]	Cross-sectional 2014	Dublin/White Caucasian	525 (300 F, 225 M)	7–13 (Mean = 10.39)	Bitter Sweet	*TAS2R38* (rs713598, rs1726866, and rs10246939) PROP/NaCl 2-sugar solutions	3-day diet history Vegetable hedonic ratings	Significant higher liking scores for cauliflower in PAV/AVI heterozygous girls compared to PAV/PAV or AVI/AVI girls *p* = 0.04 Significant higher liking for cauliflower in NTs boys compared to MTs and STs *p* = 0.03 Significant lower liking for broccoli in NTs girls compared to MTs and STs *p* = 0.02 NTs boys had a higher liking for cauliflower, while NTs girls had lower preference for broccoli Cruciferous vegetable intakes did not differ between *TAS2R38* genotype or PROP taster groups
[44]	Cross-sectional 2017	Dublin/White Caucasian	525 (300 F, 225 M)	7–13 (Mean = 10.25)	Bitter Sweet	*TAS2R38* (rs713598, rs1726866, and rs10246939) PROP/NaCl 2-sugar solutions	3-day diet history	No difference in diet quality between taster groups No significant correlations between sweet, salt, or bitter taste intensity and intake *p* > 0.05
[46]	Cross-sectional 2016	USA/136 Black, 46 White Caucasian, 2 Asians, 51 more than one ethnicity, 219 non-Hispanic	235 (124 F, 111 M)	7–14 (Mean = 10.4)	Bitter Sweet	*TAS2R38* (rs713598, rs1726866, and rs10246939) *TAS1R3* (rs35744813), *GNAT3* (rs7792845) 17-sucrose solution	Automated Self-Administered 24-h recall system	Sucrose threshold associated with bitter-sensitive Bitter-sensitive genotype had more 6% of their kcal as added sugars
[48]	Cross-sectional 2013	Dublin/White Caucasian	525 (300 F, 225 M)	7–13 (Mean = 10.25)	Bitter	*TAS2R38* (rs713598, rs1726866, and rs10246939) PROP/NaCl	3-day diet history and Frequency of eaten food	No significant differences for all nutrients or food group intakes between genotypes and phenotypes taster groups No significant difference between the proportions of taster types across “more healthful” and “less healthful” clusters of food intake, *p* = 0.06 and 0.74 for *TAS2R38* genotype and PROP taster status, respectively
[49]	6-year Follow-up 2013	USA 86% white Caucasian	73 (28 F, 45 M)	7–13 (Mean = 10.3)	Bitter	*TAS2R38* (rs713598 and rs172866) PROP/NaCl	3 24-h recalls Dutch Eating Behaviour Questionnaire	No differences in eating attitude, and the energy intake did not vary among taster groups

F = females; M = males; NT = non-tasters; MT = medium tasters; ST = supertasters; T = tasters; HP = high preference; LP = low preference; H. = high; W. weight; NW = normal weight; FFM = fat free mass; N/A = not applicable; NR = not reported. (*) Indicates studies included in the meta-analysis; (**) Condiments refers to sauces such as (shrimp paste, fish paste, fish sauce, vinegar, tomato catsup, soy sauce).

## Supplementary Materials

The following are available online at https://www.mdpi.com/2072-6643/12/7/1985/s1, Table S1. Number of papers for each taste based on phenotype or genotype classification. Table S2. Genes and SNPs associated with each taste. Table S3. Most frequently reported food based on taste qualities. Table S4. Food likes and dislikes based on taste.
