# Supplementary material for: Impact of Taste on Food Choices in Adolescence—Systematic Review and Meta-Analysis
Source: Nutrients. 2020 Jul 3;12(7):1985. doi: 10.3390/nu12071985 (PMC7400812; doi:10.3390/nu12071985)
Supplement: Supplementary file 1 [file nutrients-12-01985-s001.pdf]

nutrients

MDPI

Supplementary Table S1. Number of papers for each taste based on phenotype or genotype classification.

Taste	Phenotype	Genotype
**Bitter**	5 cohorts in 7 papers	4 cohort in 6 papers
**Sweet**	4 cohorts in 5 papers	2 studies
**Fat**	0	1 study
**Salty**	0	0
**Sour**	0	0
**Umami**	0	0

Supplementary Table S2. Genes and SNPs associated with each taste.

Taste	Gene	SNPs
**Bitter**	TAS2R38	rs713598<br>rs10246939<br>rs1726866
**Sweet**	TAS1R2	rs9701796<br>rs35874116
	TAS1R3	rs35744813
	GNAT3	rs7792845
**Fat**	CD36	rs1761667

Table 3. Most frequently reported food based on taste qualities**.**

Food categories/ Taste		**Bitter**		**Sweet**		**Fatty**	
		**Phenotype**	**Genotype**	**Phenotype**	**Genotype**	**Phenotype**	**Genotype**
Total Brassica vegetables	Pref.	0	0	0	0	0	0
	intake	0	1	0	0	0	0
Bitter green vegetables.	Pref.	0	0	0	0	0	0
	intake	1	1	0	0	0	0
Cauliflower	Pref.	2	1	0	0	0	0
	intake	0	0	0	0	0	0
Bitter gourd	Pref.	1	0	0	0	0	0
	intake	0	0	0	0	0	0
Cabbage	Pref.	3	1	0	0	0	0
	intake	0	0	0	0	0	0
Brussels sprouts	Pref.	2	1	0	0	0	0
	intake	0	0	0	0	0	0
Broccoli	Pref.	2	1	0	0	0	0
	intake	0	0	0	0	0	0
Other vegetables	Pref.	1	0	0	0	0	0
	intake	0	1	0	0	0	0
Coffee	Pref.	3	0	0	0	0	0
	intake	0	0	0	0	0	0
Dark chocolate	Pref.	1	0	0	0	0	0
	intake	0	0	0	0	0	0
Fruit	Pref.	1	0	1	0	0	0
	intake	0	1	0	0	0	0
Meats	Pref.	1	0	0	0	0	0
	intake	0	1	0	0	0	0

nutrients

MDPI

Cake	Pref.	1	0	0	0	0	0
	intake	0	1	0	0	0	0
Candy	Pref.	1	0	0	0	0	0
	intake	0	0	0	0	0	0
Donut	Pref.	1	0	0	0	0	0
	intake	0	0	0	0	0	0
Honey	Pref.	1	0	0	0	0	0
	intake	0	0	0	0	0	0
Ice cream	Pref.	1	0	0	0	0	0
	intake	0	1	0	0	0	0
Margarine	Pref.	1	0	0	0	0	0
	intake	0	0	0	0	0	0
Butter	Pref.	0	0	0	0	0	0
	intake	1	1	0	0	0	0
Fat/oil	Pref.	0	0	0	0	0	1
	intake	0	0	0	0	0	0
Mayonnaise	Pref.	2	0	0	0	0	0
	intake	0	0	0	0	0	0
Milks	Pref.	1	0	0	0	0	0
	intake	0	2	0	0	0	0
Sugar	Pref.	1	1	1	0	0	0
	intake	1	1	0	2	0	1
Salty food	Pref.	1	0	0	0	0	0
	intake	0	0	0	0	0	0
Sour food	Pref.	1	0	0	0	0	0
	intake	0	0	0	0	0	0
Chilli/spicy	Pref.	1	0	0	0	0	0
	intake	0	0	0	0	0	0

Supplementary Table S4. Food likes and dislikes based on taste**.**

**Bitter**	**Taster**	**Food-liked**	**Food-disliked**
		* Sweet-tasting food (e.g. Sugar) [28,34]<br>* Salty and sour food (e.g. condiments and sauces)<br>* Umami and fried food (meat products and fried chicken)	* Black coffee<br>* Dark chocolate<br>* Chilli peppers<br>* Cruciferous vegetables (e.g. cabbage and broccoli)
	**Non-tasters**	**Food-liked**	**Food-disliked**
		* Cruciferous vegetables (e.g. brussels sprouts and cauliflower)	Nothing reported
